# Supplementary material for: A Global Screen for Assembly State Changes of the Mitotic Proteome by SEC-SWATH-MS
Source: Cell Syst. 2020 Feb 26;10(2):133–155.e6. doi: 10.1016/j.cels.2020.01.001 (PMC7042714; doi:10.1016/j.cels.2020.01.001)

Q9Y6X8 | ZHX2_HUMAN | ZHX2 AFR1 KIAA0854 RAF
Monomer MW [kDa]: 92.307 Monomer expected elution fraction: 41

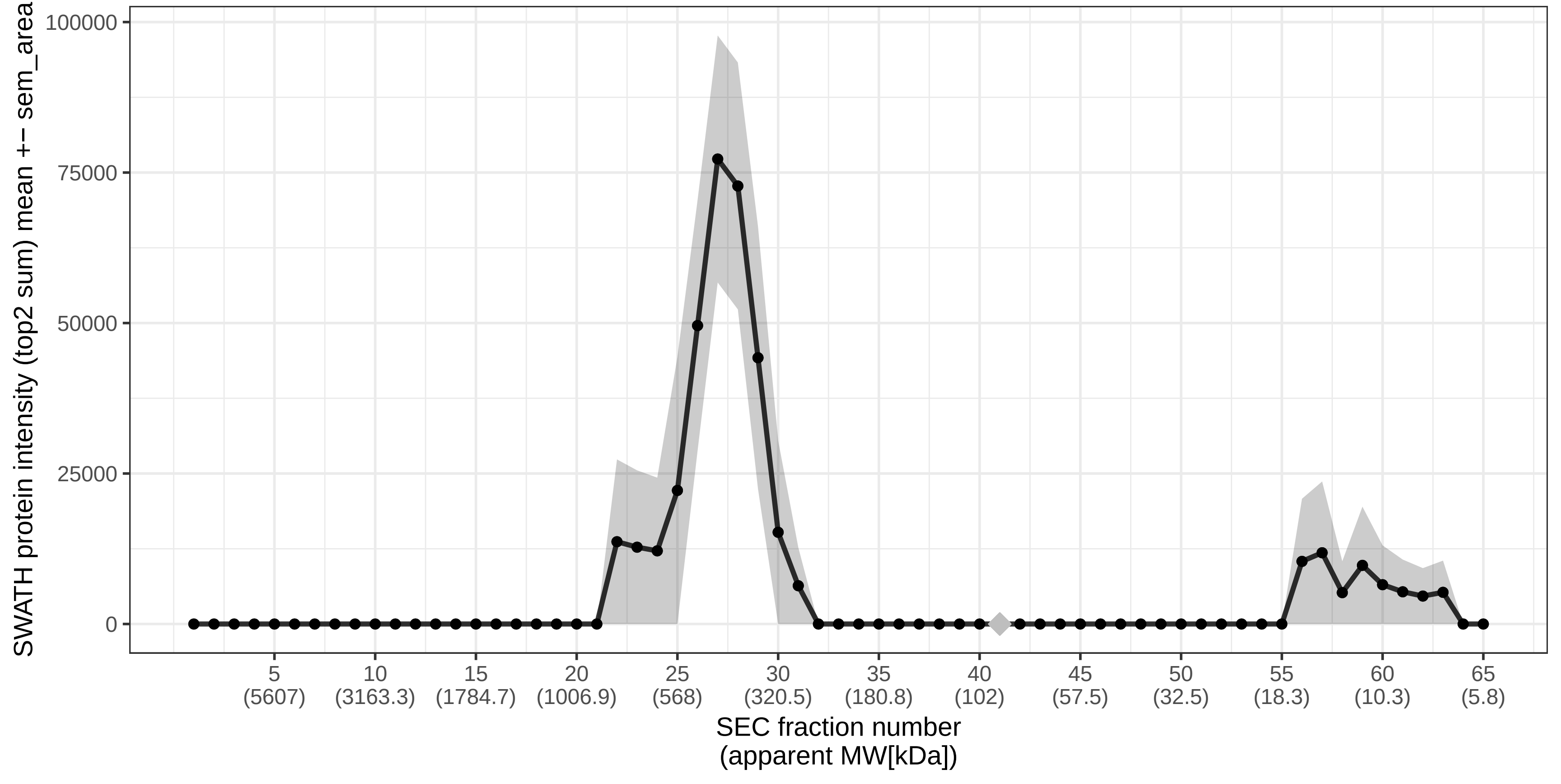

Supplement: Data S1. SEC-SWATH-MS Protein Chromatograms, Related to Figure 1 [file mmc6.zip › SECchrom_Q9Y6X8_ZHX2_HUMAN_ZHX2_AFR1_KIAA0854_RAF.pdf]
